# Genome-wide identification of long non-coding RNA and mRNA profiling using RNA sequencing in subjects with sensitive skin

**DOI:** 10.18632/oncotarget.23147

**Published:** 2017-12-12

**Authors:** Li Yang, Lechun Lyu, Wenjuan Wu, Dongyun Lei, Ying Tu, Dan Xu, Jiaqi Feng, Li He

**Affiliations:** ^1^ Department of Dermatology, The First Affiliated Hospital of Kunming Medical University, Kunming, China; ^2^ Technology Transfer Center, Kunming Medical University, Department of Physiology, Kunming Medical University, Kunming, China

**Keywords:** sensitive skin, lncRNA, mRNA, RNA sequencing

## Abstract

Sensitive skin (SS) is a condition of subjective cutaneous hyper-reactivity. The role of long non-coding RNAs (lncRNAs) in subjects with SS is unclear. Therefore, the aim of the present study was to provide a comprehensive profile of the mRNAs and lncRNAs in subjects with SS. Gene Ontology (GO) and Kyoto Encyclopedia of Genes and Genomes (KEGG) analysis presented the characteristics of associated protein-coding genes. In addition, a co-expression network of lncRNA and mRNA was constructed to identify potential underlying regulation targets; the results were verified by quantitative real-time PCR (qRT-PCR) and RNA-seq analyses in patients with SS and normal samples. Compared with the normal skin group, 266 novel lncRNAs and 6750 annotated lncRNAs were identified in the SS group. A total of 71 lncRNA transcripts and 2615 mRNA transcripts were differentially expressed (*P* < 0.05). The heat signature of the SS samples could be distinguished from the normal skin samples, whereas the majority of the genes that were present in enriched pathways were those that participated in focal adhesion, PI3K-Akt signaling, and cancer-related pathways. Five transcripts were selected for qRT-PCR analysis and the results were consistent with RNA-seq. The results suggested that LNC_000265 may play a role in the epidermal barrier structure of patient with SS. The data suggest novel genes and pathways that may be involved in the pathogenesis of SS and highlight potential targets that could be used for individualized treatment applications.

## INTRODUCTION

Sensitive skin (SS) is a broad term used to describe a multitude of clinical findings that are attributed to different sensory perceptions, namely facial irritation, burning, stinging, tightness, tingling, pain, and pruritus [1]. The sensitive skin syndrome (SSS) is considered as a state of hyperactivity to specific environmental stimuli that is caused from a single and/or a number of underlying pathologies [2]. The main disadvantage encountered during the diagnosis of the disease is the lack of an objective-screening test [3]. The complex nature of the skin disease syndrome requires the use of a diagnostic algorithm and the need to test patients with multiple patch testing, prior to the establishment of a definite diagnosis, as it has been shown from the lack of association between different allergens in subjects with positive allergic reactions with regard to each allergen alone (SDS and/or lactic acid) [3, 4]. Nevertheless, several studies have suggested a link between SS and disruption of the epidermal barrier function, resulting in the perception of skin discomfort [5, 6]. Despite these promising findings, the molecular network that contributes to the development of SS has not been elucidated to date.

Long non-coding RNAs (lncRNAs) are a class of RNA sequences that are more than 200 nt in length and are involved in the regulation of translation process, although they do not possess protein coding potential [7]. A multitude of studies have shown that lncRNAs are involved in the regulation of developmental processes and in the progression of several human diseases [8–11], while their expression and localization varies among different cell types and subcellular compartments [12–17]. LncRNAs have been found crucial to genomic imprinting, dosage compensation, and pluripotency-regulation [18, 19]. The rapid progress of RNA sequencing (RNA-seq) promoted the exploration and research of non-coding RNAs, and novel lncRNAs have been identified by different pipelines using RNA-seq data [20, 21]. RNA-seq exhibits several advantages compared to the previously established methodologies that have been used for the evaluation of the complete set of transcripts in the cell, such as hybridization-based approaches and specialized microarrays. RNA-Seq has been successfully used to provide a ‘digital measurement’ of the gene expression difference between different tissues [22]. Although no reports on the contribution of lncRNA in the development of SS have been reported, it has been suggested that these RNA sequences play a significant role in skin homeostasis and related skin diseases [23, 24]. Several studies demonstrated the involvement of lncRNAs in the differentiation and maintenance of normal human keratinocytes and epidermal tissues [25, 26]. For example, lncRNA such as anti-differentiation non-coding RNA (ANCR) and terminal differentiation-induced non-coding RNA (TINCR) are vital for epidermal stability [27, 28]. Sonkoly *et al*. identified a novel lncRNA, namely psoriasis susceptibility-related RNA gene induced by stress (PRINS) that is involved in the susceptibility to psoriasis [29]. The authors suggested that PRINS may play an important role in psoriasis by evidence derived from psoriasis patients and *in vitro* cell culture experiments [29]. The use of bioinformatics methods has been adopted in the investigation of the genes involved in the development of atopic dermatitis and psoriasis. Notably, differentially expressed genes (DEGs) were associated with epidermis development and immune response in atopic dermatitis [30]. Similarly, enrichment analysis of psoriasis- correlated modules revealed that pathways involved in short chain fatty acid metabolism, olfactory signaling, and regulation of leukocyte-mediated cytotoxicity were the main pathways in which the DEGs were identified [31]. Of note is that more than 50% of the co-expressed genes in 18 psoriasis patients and 16 healthy controls were lncRNAs [31].

In view of the significant roles of lncRNA in the regulation and the differentiation of epidermal homeostasis and the disruption of the epidermal barrier function in SS, we hypothesized that lncRNAs may also take part in the pathogenesis of SS. The aim of the present study was to provide a more comprehensive and validated conclusion regarding the identification of differentially expressed lncRNAs in SS tissues.

## RESULTS

### Overview of RNA-Seq and mRNAs and lncRNAs identification

**Figure 1:**
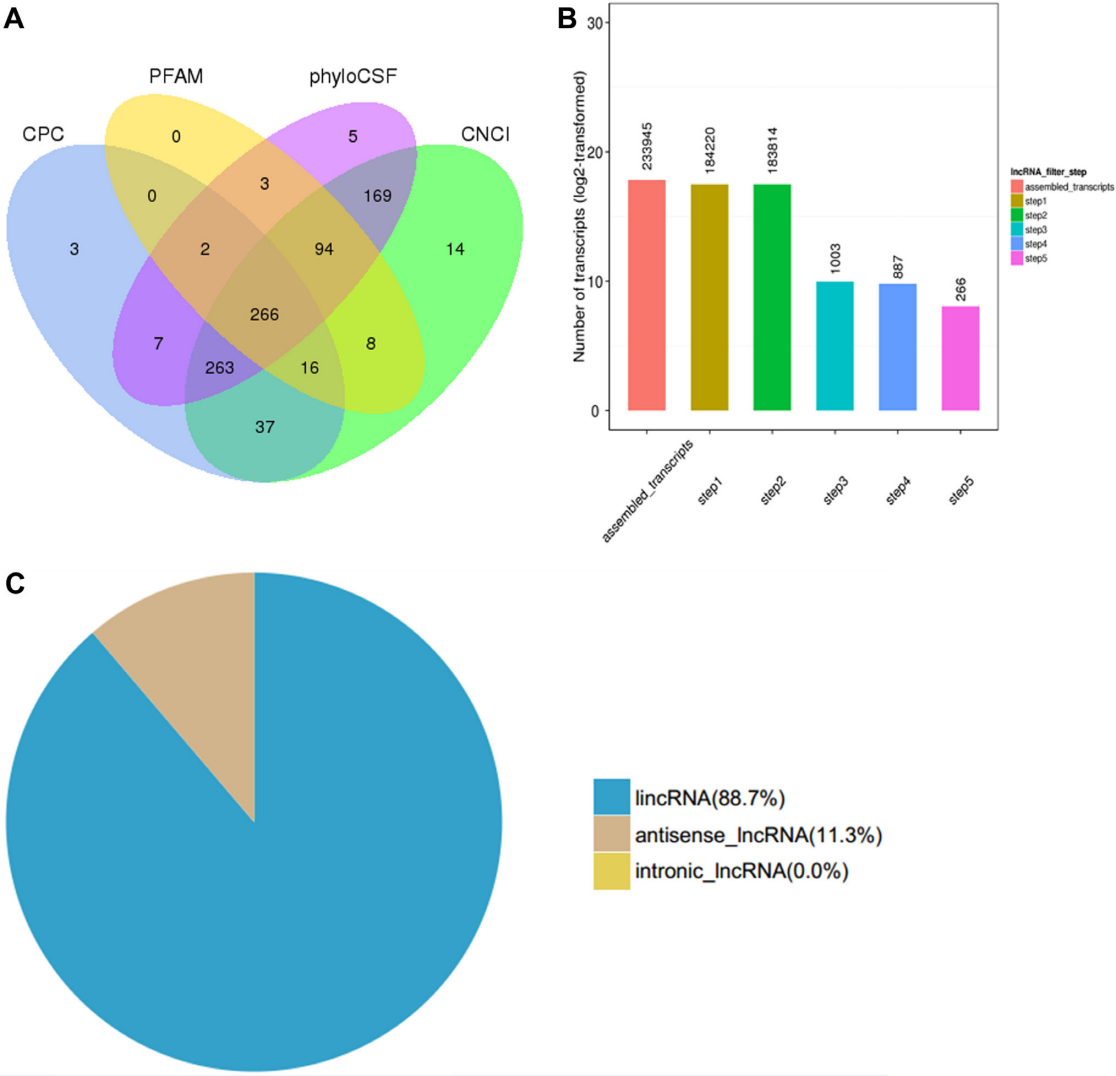
Transcriptome analysis of lncRNA by RNA-seq in three skin samples of subjects with SS and three normal skin samples (**A**) Venn diagram of screening results. The sum of the numbers in each large circle represents the total number of non-coding transcripts of the software, and the overlapping parts of the circle represent the non-coding transcripts common to the software. Fragments per kilobase of transcript sequence per millions base pairs sequenced (FPKM), phylogenetic coding substitution frequency (PhyloCSF), coding potential calculator (CPC) and coding-non-coding-index (CNCI). (**B**) The abscissa represents the screening step and the ordinate represents the corresponding steps after screening the number of transcripts. (**C**) Pie chart distribution of novel lncRNAs identified based on antisense and intronic forms.

The RefSeq (Build 37.3) and the GENCODE v19 databases were selected as the annotation references for mRNA and lncRNA analyses, respectively. Tissues from three patients with SS and three normal skin tissues were used to construct the RNA-seq library. The reads were mapped using Cufflinks and this resulted in 233,945 assembled transcripts. The number of transcripts corresponding to more than two exons were selected in order to filter abundant of low-expression, low-confidence single exon transcripts. In addition, the transcripts with a length of >200 bp were selected and the known functional genes were removed. The transcripts that contained an exon area overlapping with the annotation database were screened by the Cuffcompare function. The lncRNAs with overlapping exon areas assembled in the database were included as annotation lncRNA database into subsequent analysis. Furthermore, the expression of each transcript was calculated by Cuffquant, and the transcripts with expected number of fragments per kilobase of transcript sequence per millions base pairs sequenced (FPKM) ≥0.5 were selected. This selection process yielded 183,814 filtered transcript isoforms. Finally, the protein-coding transcripts were excluded by Phylogenetic Coding Substitution Frequency (PhyloCSF), PfamScan (v1.3), Coding Potential Calculator (CPC), and Coding-Non-Coding-Index (CNCI) [21]. The transcripts identified by the four methods were deemed as confidently expressed lncRNAs. The final screen included a coding potential score lower than 0 (CPC < 0), CNCI and PLEK scores lower than 0 (<0) and Pfam and E-values lower than 0.001 (<0.001). The final results yielded 887 novel assembled lncRNAs that corresponded to 266 novel lncRNA transcripts (Figure 1A, 1B), including 236 (88.7%) lncRNAs and 30 (11.3%) antisense lncRNAs (Figure 1C).

**Figure 2:**
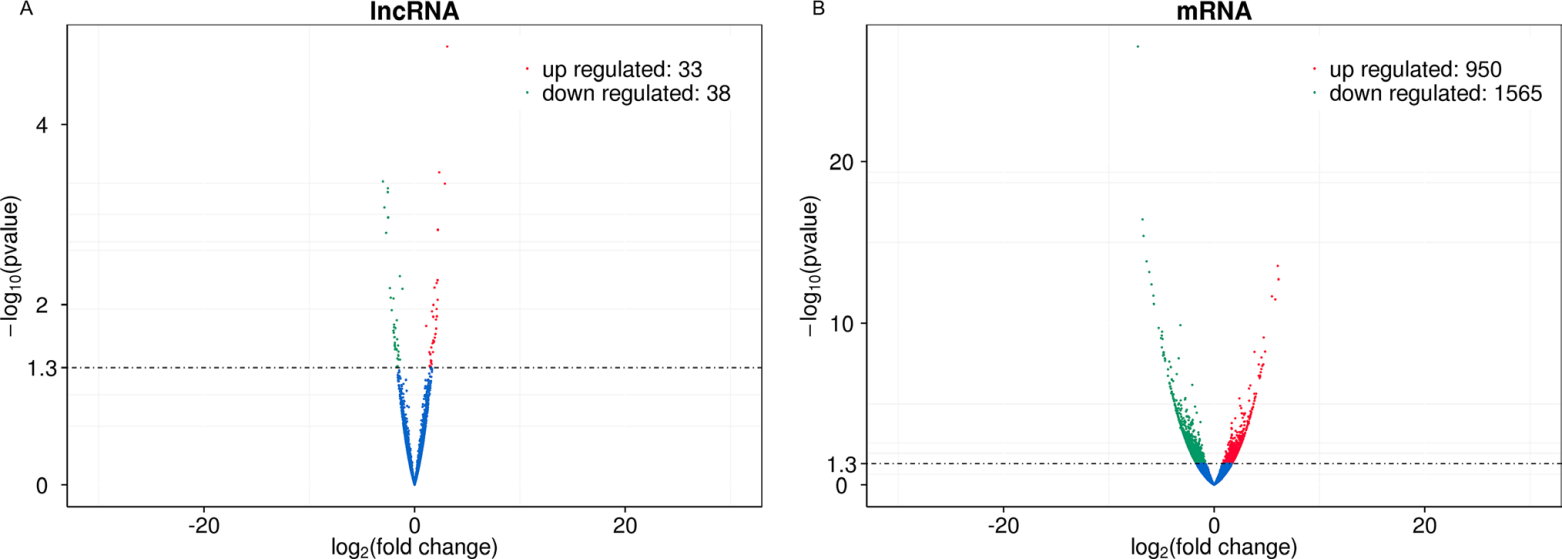
Volcano plots of DE transcripts The difference of lncRNAs expression profiles (**A**) and mRNAs expression profiles (**B**) can be noted in the overall distribution of the transcripts. The red points in the plot represent upregulated transcripts and the green points represent downregulated transcripts. The filter threshold is *p* value < 0.05 by default.

**Figure 3:**
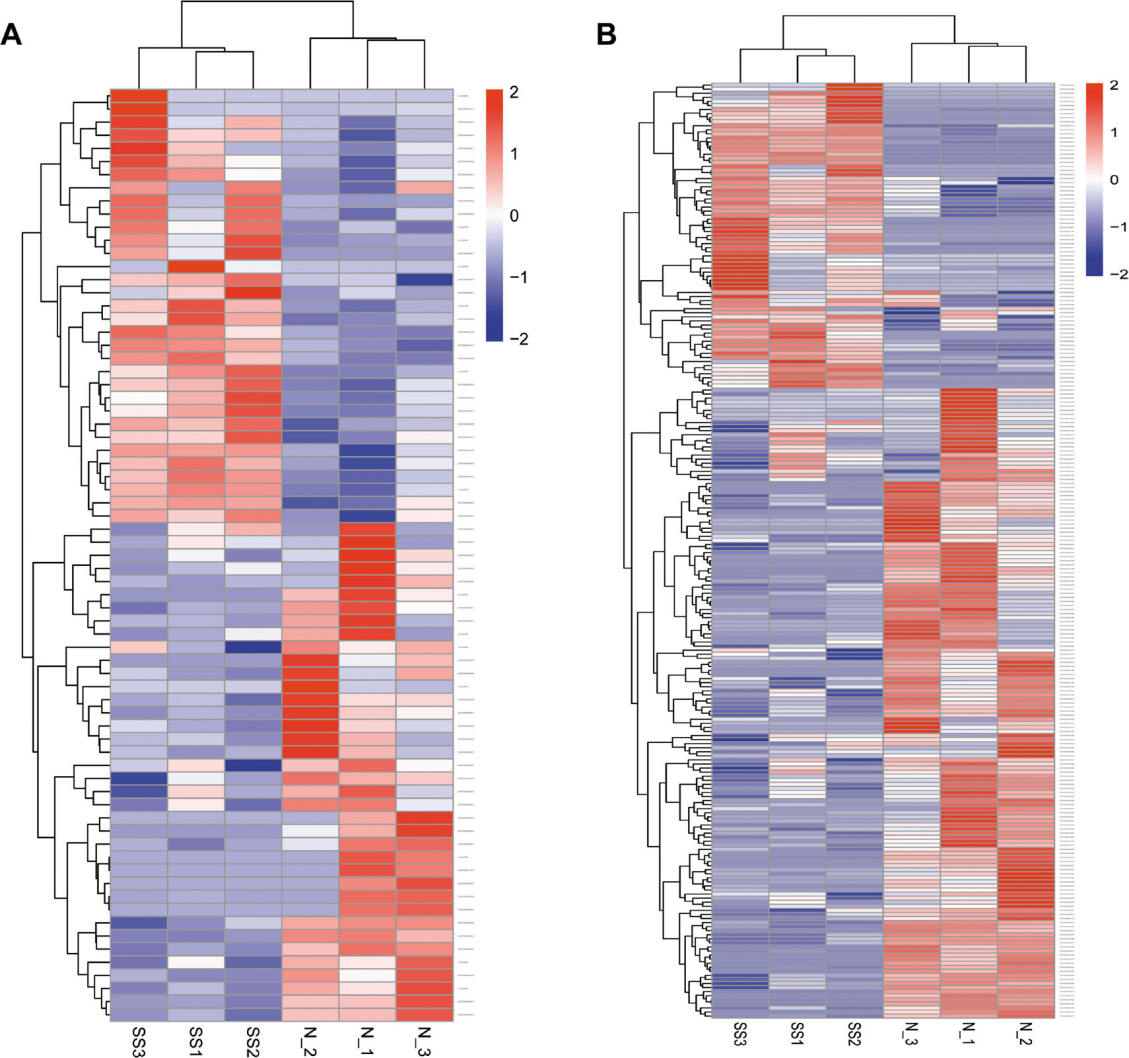
Hierarchical heat maps of the differentially expressed profiles between sensitive and normal skin lncRNA (**A**) and mRNA (**B**) were used for analysis of the gene expression data, for which the cluster analysis arranges samples into groups according to their expression levels. Each column represents a sample and each row represents a transcript. ‘Red’ indicates higher expression, and ‘blue’ indicates lower expression. Sensitive skin (SS), normal skin (N).

A total of 6750 annotated lncRNAs, 2718 (40.27%) antisense lncRNAs, 2251 (33.35%) lncRNAs, and 542 (8.00%) intronic RNAs were identified (Supplementary Table 1). In the skin tissue of patient with SS, 71 lncRNA transcripts (33 up-regulated and 38 down-regulated) and 2615 mRNA transcripts (950 up-regulated and 1565 down-regulated) were differentially expressed compared with the normal skin (Figure 2A, 2B). The differentially expressed lncRNAs and mRNAs that were previously selected were further screened by heat maps between the SS patients groups and the normal groups using unsupervised hierarchical clustering analysis (Figure 3A, 3B). The analysis revealed that the SS samples could be distinguished from the normal samples as a different heat signature was evident in each case (Figure 3A, 3B). It is interesting to note that this discrimination could be achieved by both lncRNA and/or mRNA screening, whereas the heat signatures were very similar in both cases with regard to the up-regulated and down-regulated genes (Figure 3A, 3B). The top 20 differentially expressed mRNA and lncRNAs are listed in Table 1 and 2.

**Table 1:** Top 20 differentially expressed mRNAs with >1.5-fold change in 3 sensitive skin (SS) compared with normal skin tissue (*N*)

Upregulated mRNAs			Downregulated mRNAs		
Transcript ID (Ensembl_Gene_ID)	*P*-value	Fold change (SS vs. *N*)	Transcript ID (Ensembl_Gene_ID)	*P*-value	Fold change (SS vs. *N*)
ENST00000502213 (TLR1)	1.92E-13	6.11601	ENST00000557022 (ZFP36L1)	1.96E-12	−5.76884
ENST00000283684 (ATP8B1)	2.94E-14	6.04138	ENST00000616053 (TCF4)	6.53E-12	−5.72168
ENST00000620565 (UHRF1)	3.42E-12	5.80807	ENST00000502252 (CORIN)	2.00E-10	−5.27057
ENST00000510411 (HNRNPH1)	2.18E-12	5.48796	ENST00000535987 (FOS)	8.45E-10	−5.0481
ENST00000336665 (AGAP1)	5.80E-09	4.84147	ENST00000417268 (SCAF8)	5.84E-10	−4.97343
ENST00000621364 (NOMO2)	7.76E-10	4.70092	ENST00000602390 (COMMD3-BMI1)	3.10E-09	−4.9622
ENST00000347088 (YY1AP1)	3.58E-08	4.67080	ENST00000264266 (MFSD1)	3.32E-10	−4.94658
ENST00000541717 (MELK)	4.46E-08	4.56854	ENST00000442173 (DOCK9)	9.59E-10	−4.8981
ENST00000450331 (PNPLA6)	1.31E-08	4.49646	ENST00000613065 (ZNF254)	9.90E-09	−4.84424
ENST00000360661 (BAK1)	6.78E-08	4.48298	ENST00000514633 (HNRNPAB)	6.47E-09	−4.81581
ENST00000399202 (FAM214A)	1.08E-07	4.42187	ENST00000520492 (ZFPM2)	8.54E-09	−4.80934
ENST00000380989 (PITRM1)	2.01E-07	4.36727	ENST00000614805 (PLXNB2)	9.12E-09	−4.7907
ENST00000396499 (CCDC125)	1.78E-07	4.34897	ENST00000532891 (ARHGAP27)	6.45E-09	−4.78249
ENST00000506339 (HNRNPAB)	2.36E-07	4.32278	ENST00000503781 (PIEZO2)	1.46E-08	−4.69927
ENST00000523714 (ANXA6)	2.51E-07	4.318563	ENST00000383083 (PLSCR4)	2.14E-08	−4.66819
ENST00000368488 (CEP85L)	3.63E-08	4.23751	ENST00000283921 (HOXA10)	1.68E-08	−4.64941
ENST00000342788 (ERBB4)	1.81E-07	4.22746	ENST00000526927 (LTBP3)	1.84E-07	−4.39143
ENST00000577278 (AXIN2)	2.27E-06	3.98945	ENST00000325468 (GYLTL1B)	7.09E-08	−4.38332
ENST00000382422 (BAZ1A)	3.47E-06	3.92167	ENST00000251268 (MEGF8)	2.39E-08	−4.25831
ENST00000412006 (GABBR1)	3.52E-06	3.90662	ENST00000355973 (CCDC77)	4.83E-07	−4.24682

*P*-values < 0.05 were considered significant.

**Table 2:** Top 20 differentially expressed lncRNAs with >1.5-fold change in 3 sensitive skin (SS) compared with normal skin tissue (*N*)

Upregulated lncRNAs			Downregulated lncRNAs		
Transcript ID (Gene_Symbol)	*P*-value	Fold change (SS vs. *N*)	Transcript ID (Gene_Symbol)	*P*-value	Fold change (SS vs. *N*)
ENST00000624863.1 (AC003973.3)	1.36E-05	3.10833	LNC_000101	0.000429	−3.0039
ENST00000585940.1 (CTD-2537I9.12)	0.000452	2.87593	ENST00000589456.1 (CTD-2537I9.12)	0.000831	−2.86602
ENST00000441722.5 (ZFAS1)	0.000339	2.33685	ENST00000457658.5 (TTTY15)	0.001591	−2.70333
ENST00000438107.1 (RP11-706O15.3)	0.001474	2.20917	ENST00000564363.1 (RP11-1100L3.8)	0.000563	−2.5488
ENST00000449766.5 (AC016735.2)	0.008838	2.19405	ENST00000564531.1 (RP11-1100L3.8)	0.00051	−2.53449
LNC_000156	0.005353	2.17926	ENST00000528549.1 (TBX5-AS1)	0.001077	−2.51396
LNC_000208	0.013481	2.12202	ENST00000567257.5 (LOXL1-AS1)	0.006557	−2.34528
LNC_000082	0.011171	2.09752	LNC_000265	0.008368	−2.26627
ENST00000607746.1 (LINC01215)	0.005734	2.09382	ENST00000566193.1 (RP11-424G14.1)	0.011503	−2.17116
LNC_000244	0.018443	2.04553	ENST00000331787.2 (TTTY14)	0.01933	−2.0051
LNC_000144	0.014573	2.03612	ENST00000414790.5 (H19)	0.008537	−1.99685
ENST00000424210.1 (SPAG5-AS1)	0.021094	1.98796	ENST00000503474.5 (HAND2-AS1)	0.020475	−1.9804
ENST00000381108.3 (RP11-706O15.3)	0.021268	1.974543	ENST00000439725.5 (H19)	0.017798	−1.95243
ENST00000562127.1 (RP11-445O3.3)	0.023283	1.91734	ENST00000507362.5 (RP11-319G6.1)	0.016665	−1.9427
ENST00000500989.2 (LINC00861)	0.006481	1.89280	ENST00000553510.1 (RP11-293M10.1)	0.023008	−1.92564
ENST00000559298.5 (IQCH-AS1)	0.025628	1.84106	LNC_000084	0.022983	−1.89912
ENST00000414002.5 (SNHG5)	0.010083	1.78812	LNC_000008	0.027705	−1.88524
ENST00000355358.1 (GATA3-AS1)	0.013621	1.76931	ENST00000567089.1 (RP11-265N6.2)	0.028664	−1.88521
LNC_000165	0.026313	1.76915	ENST00000620503.1 (RP11-115H13.1)	0.027862	−1.88198
ENST00000417483.5 (RP11-557H15.3)	0.025022	1.76723	LNC_000087	0.026409	−1.87013

### Genomic features of lncRNAs

**Figure 4:**
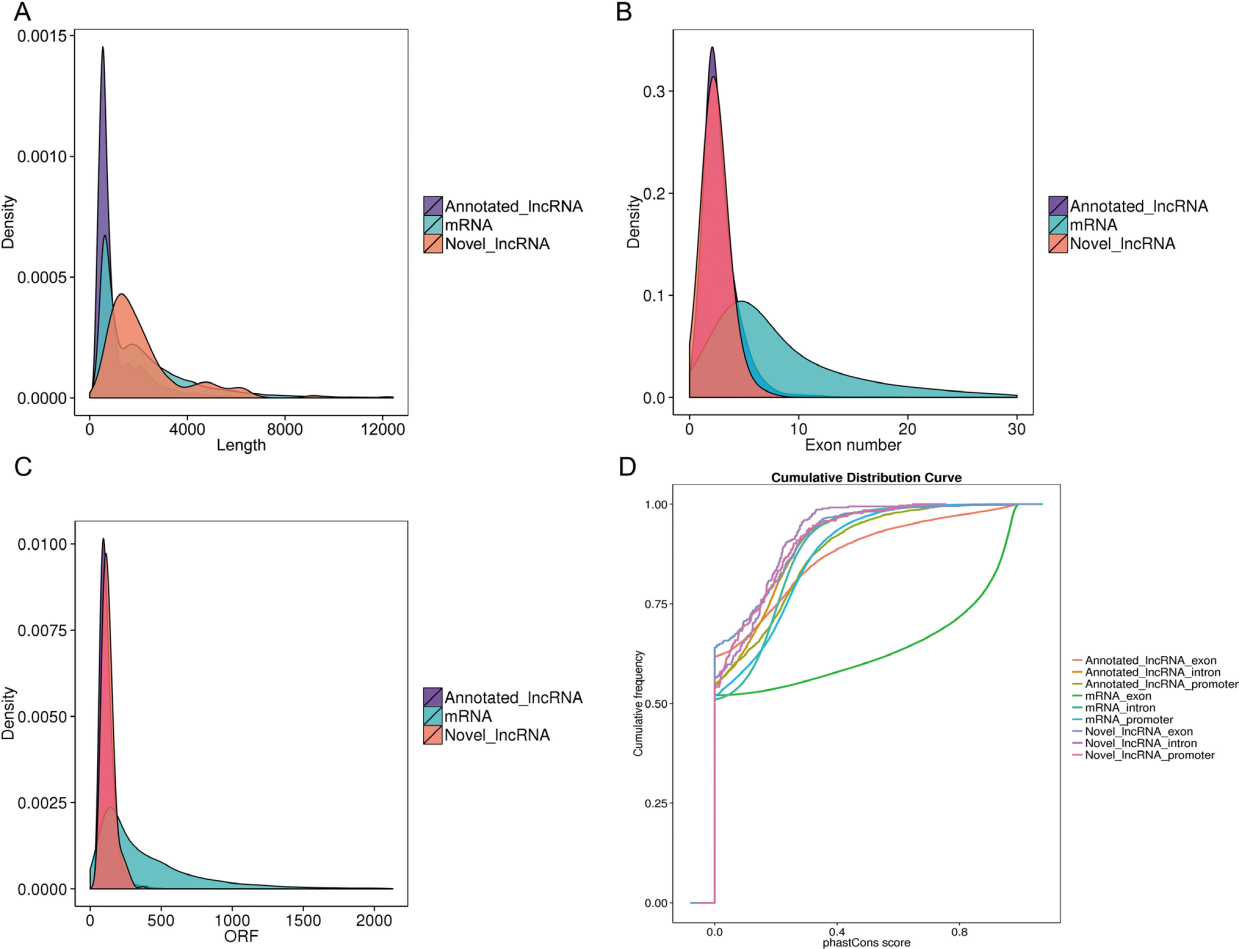
Genomic features of predicted lncRNAs (**A**) Length distribution of coding transcripts (blue), known lncRNAs (purple), and new predicted lncRNAs (red). (**B**) Exon number distribution of coding transcripts and lncRNAs. (**C**) Orf length distribution of coding transcripts and lncRNAs. (**D**) Conservation score for coding transcripts and lncRNAs as determined by the phastCons software.

The characteristics of the lncRNAs and their comparison with protein-coding genes were further examined. The predicted novel lncRNA were greater in length (Figure 4A) and contained fewer exons (Figure 4B) compared with the mRNA transcripts. Furthermore, we identified that lncRNAs were shorter in ORF length (Figure 4C) and appeared less conserved than mRNA transcripts (Figure 4D). These findings were in agreement with previous studies [32]. The predicted lncRNA transcripts indicated different genomic characteristics compared with protein coding transcripts.

### Alternative splicing events

**Figure 5:**
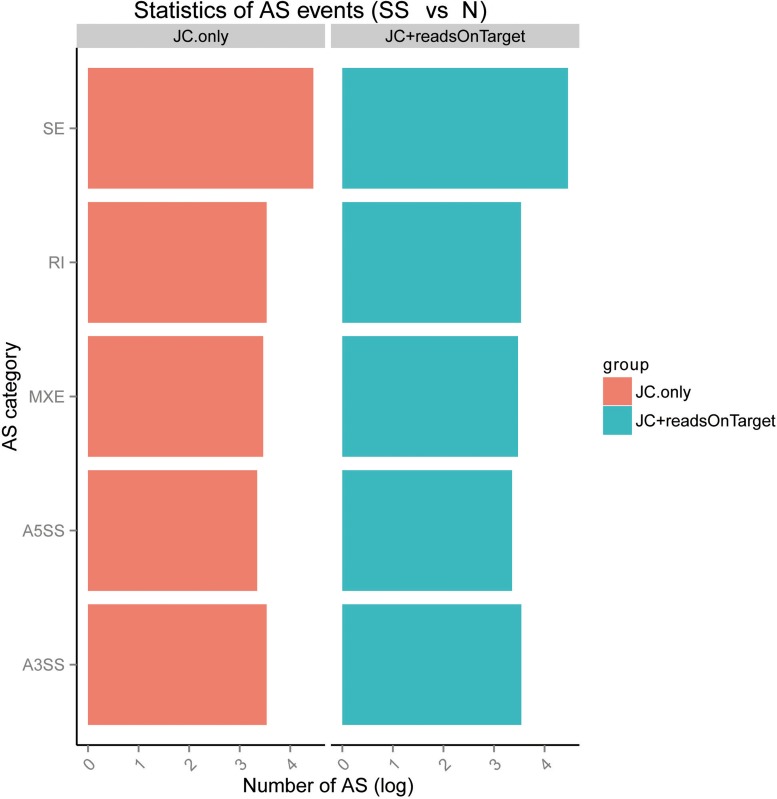
Expression amounts of alternative splicing (AS) events and differential analysis using rMATS (http://rnaseq-mats.sourceforge.net/index.html) SE: skipped exon; MXE: mutually exclusive exons; A5SS: alternative 5′ splice site; A3SS: alternative 3′ splice site; RI: retained intron.

We calculated the expression amounts of AS events. The results are shown in Figure 5 and Table 3. The results showed that AS events are involved in SS.

**Table 3:** The type and quantitative statistic analysis of AS

EventType	NumEvents.JC	SigEvents.JC		
		Total	Upregulated	Downregulated
SE	28594	27	11	16
MXE	2902	3	3	0
A5SS	2211	2	1	1
A3SS	3402	2	1	1
RI	3406	18	0	18

*P*-value < 0.05 were considered significant

### GO and KEGG pathway analysis

**Figure 6:**
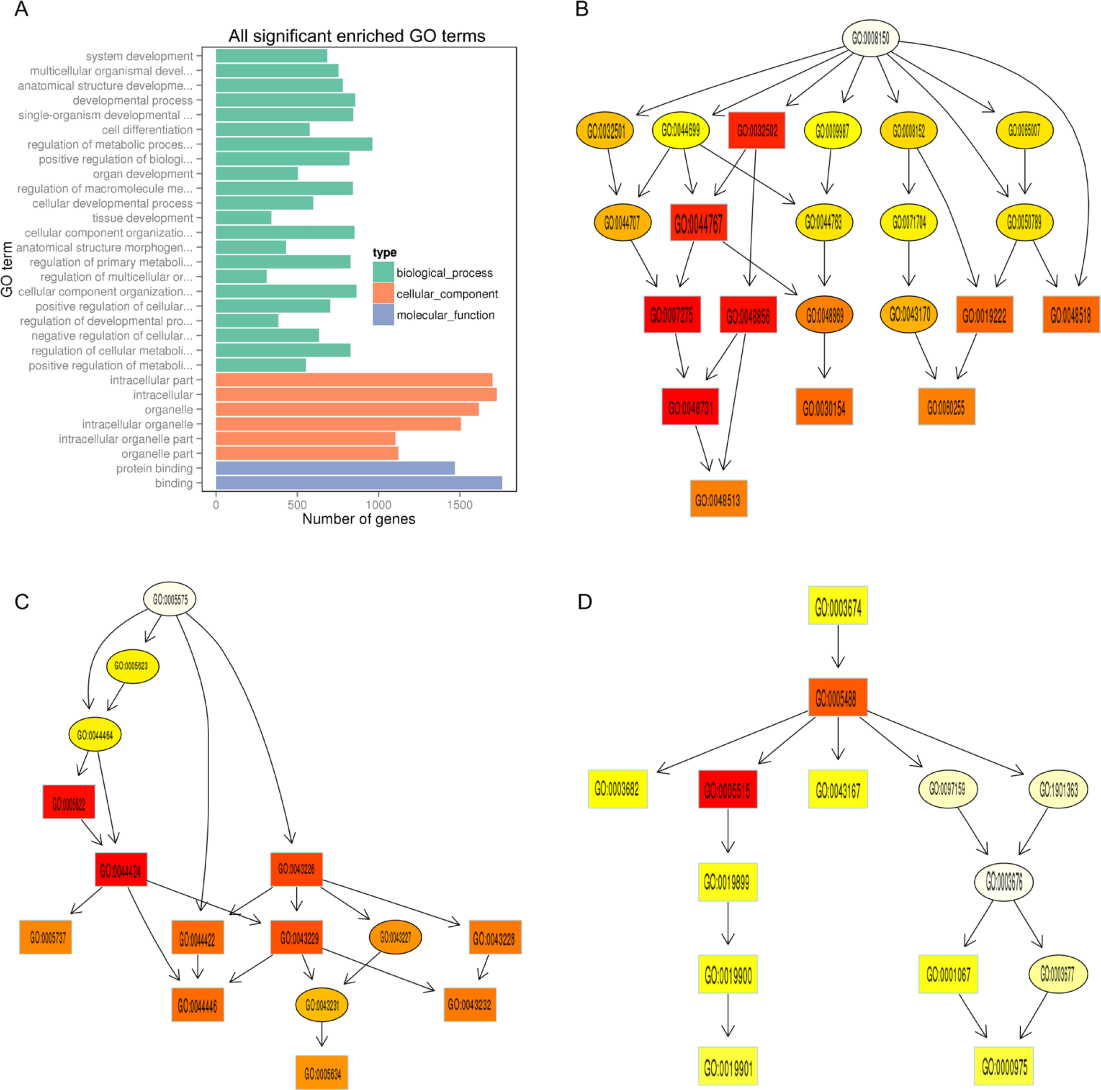
GO enrichment analysis of differentially expressed protein-coding genes (**A**) The ordinate represents the next level GO term of the three categories of GO. The abscissa represents the gene number under the term. In the directed acyclic graph (DAG) the downstream term corresponds to a subset of the upstream term. The depth of the color represents the degree of enrichment (**B–D**). The DAG of over-represented biological process (B), cellular component (C), and molecular function (D) terms in GO analysis between sensitive skin and normal skin.

To establish the function and connection of differentially expressed mRNAs, GO and KEGG pathway enrichment analysis were conducted. In the GO analysis, the apparently enriched terms are shown in Figure 6A and Supplementary Table 2. The gene networks were mainly associated with the following biological process terms: system development, multicellular organism development, anatomical structure development, and developmental process, whereas with regard to the cellular component terms the networks appeared to be active both in the intracellular part and the organelles (Figure 6A). The main molecular functions involved in the gene networks were protein binding and binding (Figure 6A).

Directed acyclic graphs (DAGs) were constructed in order to highlight the associations among the enriched GO terms (Figure 6B–6D), in which the downstream terms are subsets of the upstream terms. The GO analysis demonstrated that KRT27 and CLDN5 genes, which are associated with keratinocyte differentiation and epidermal development, were highly expressed in the SS GO term “system development”. In addition, IL-27RA and CCL18, which are key cytokine and chemokine genes involved in inflammation, were specifically expressed in the SS GO term “protein binding and binding” (Figure 6A).

**Figure 7:**
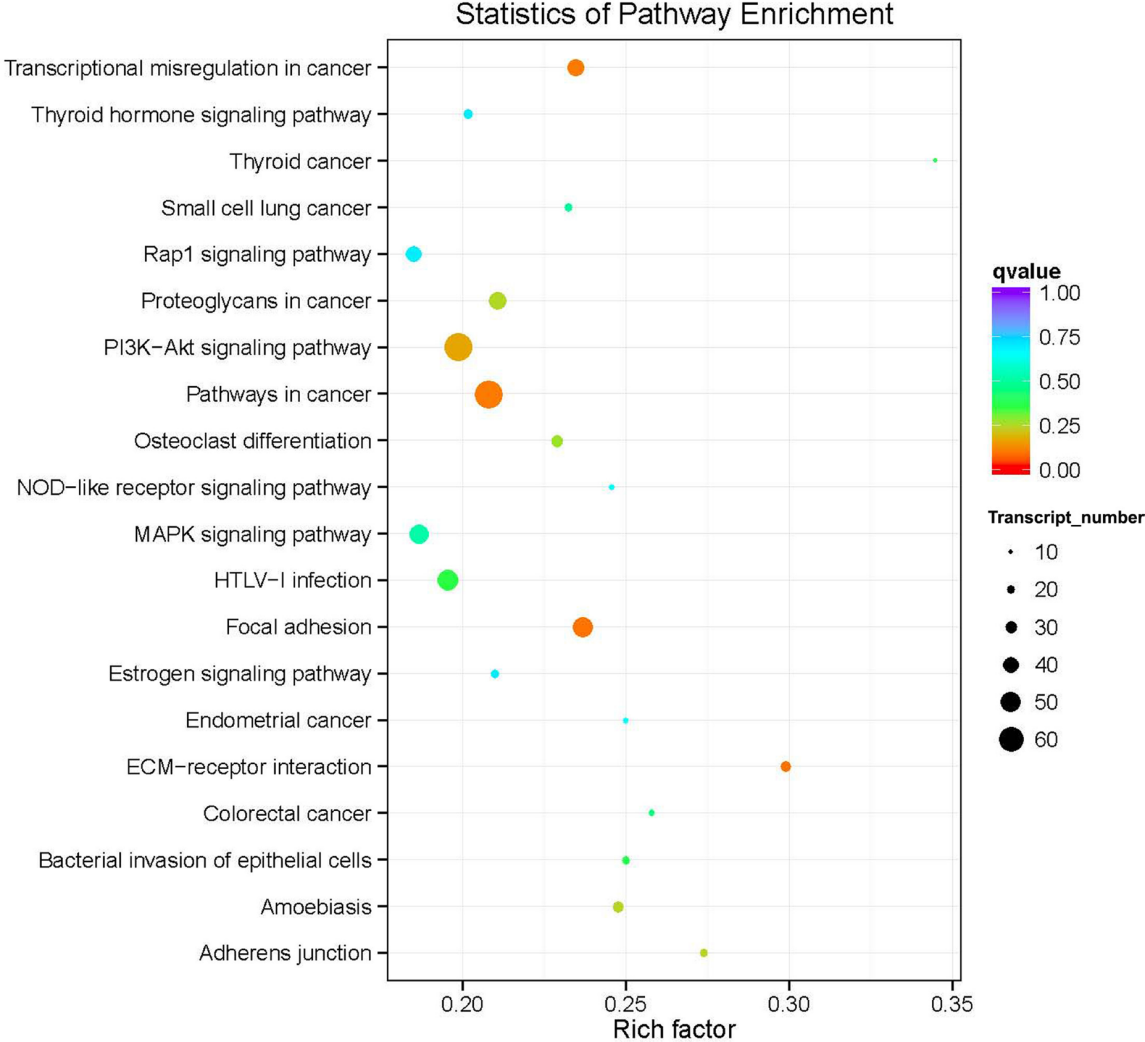
Top 20 over-represented KEGG pathways of the common differentially expressed genes The color coding (red, orange, green, light blue, purple) represents the *q* value i.e. the lowest false discovery rate (FDR), whereas the size of the dots represents the number of the genes involved in each pathway.

KEGG pathway analysis deduces the systematic biological behavior by mapping the protein-coding DEGs to the KEGG reference pathway. The results indicated that the transcripts with the lowest *q* value (i.e. lowest false discovery rate FDR) were involved in transcriptional misregulation in cancer, cancer-related pathways, focal adhesion, and ECM receptor interactions (Figure 7). Among these, the highest number of genes involved was reported for the cancer-related pathways (Figure 7). A considerable high number of genes were enriched into the PI3K-Akt signaling pathway, although the *q* value was somewhat higher than that noted for the aforementioned biological processes (Figure 7).

### qRT-PCR validation

**Figure 8:**
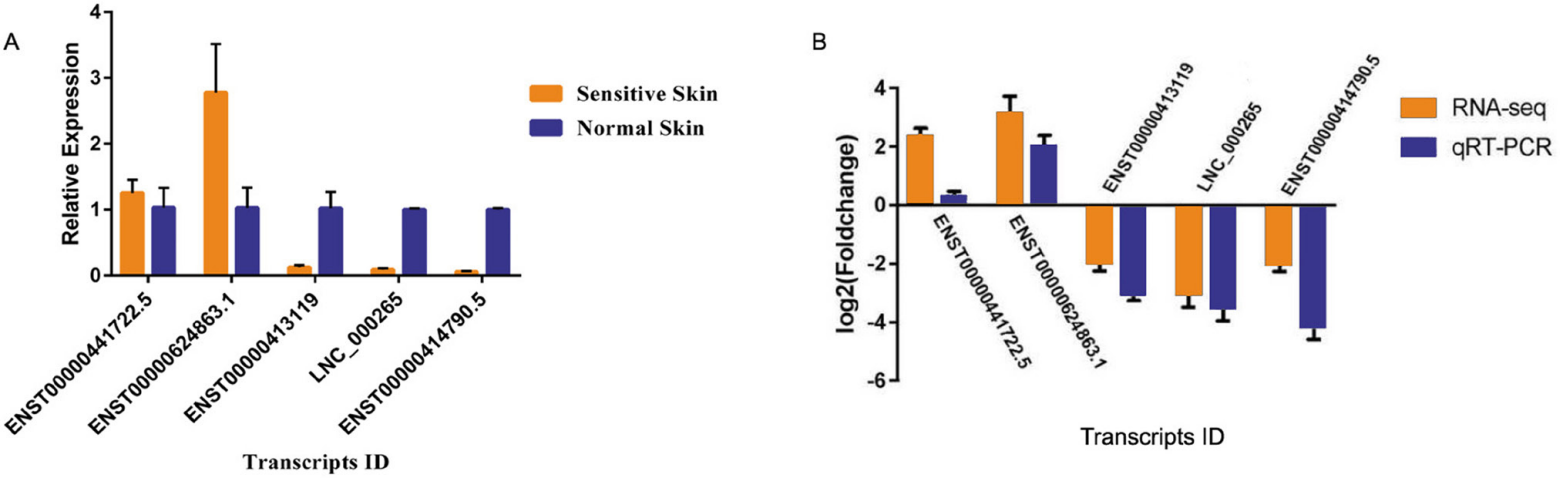
Relative expression analysis of candidate mRNA and lncRNAs in sensitive and normal skin tissues, as determined by qRT-PCR (**A**) Validation of five candidate transcripts in 13 sensitive skin and normal skin tissues. The results were consistent. (**B**) The heights of the columns represent the log2 (fold changes). The trends of the qRT-PCR results were consistent with the RNA-seq data.

We randomly selected five transcripts (LNC_000265, ENST00000441722.5, ENST00000414790.5, ENST000 00624863.1, and ENST00000413119) for qRT-PCR in 10 SS and 10 normal skin samples in order to validate the data derived by RNA-seq. The results yielded a similar trend with regard to up- and/or down-regulation for each transcript compared with the RNA-Seq data. With regard to the up-regulated transcripts, RNA-seq appeared to be more sensitive compared with qRT-PCR (Figure 8). The data of the qRT-PCR assays were in agreement with the RNA-seq results and confirmed the reliability and the validity of the RNA-Seq analysis (Figure 8).

### Predicted function of lncRNA

**Figure 9:**
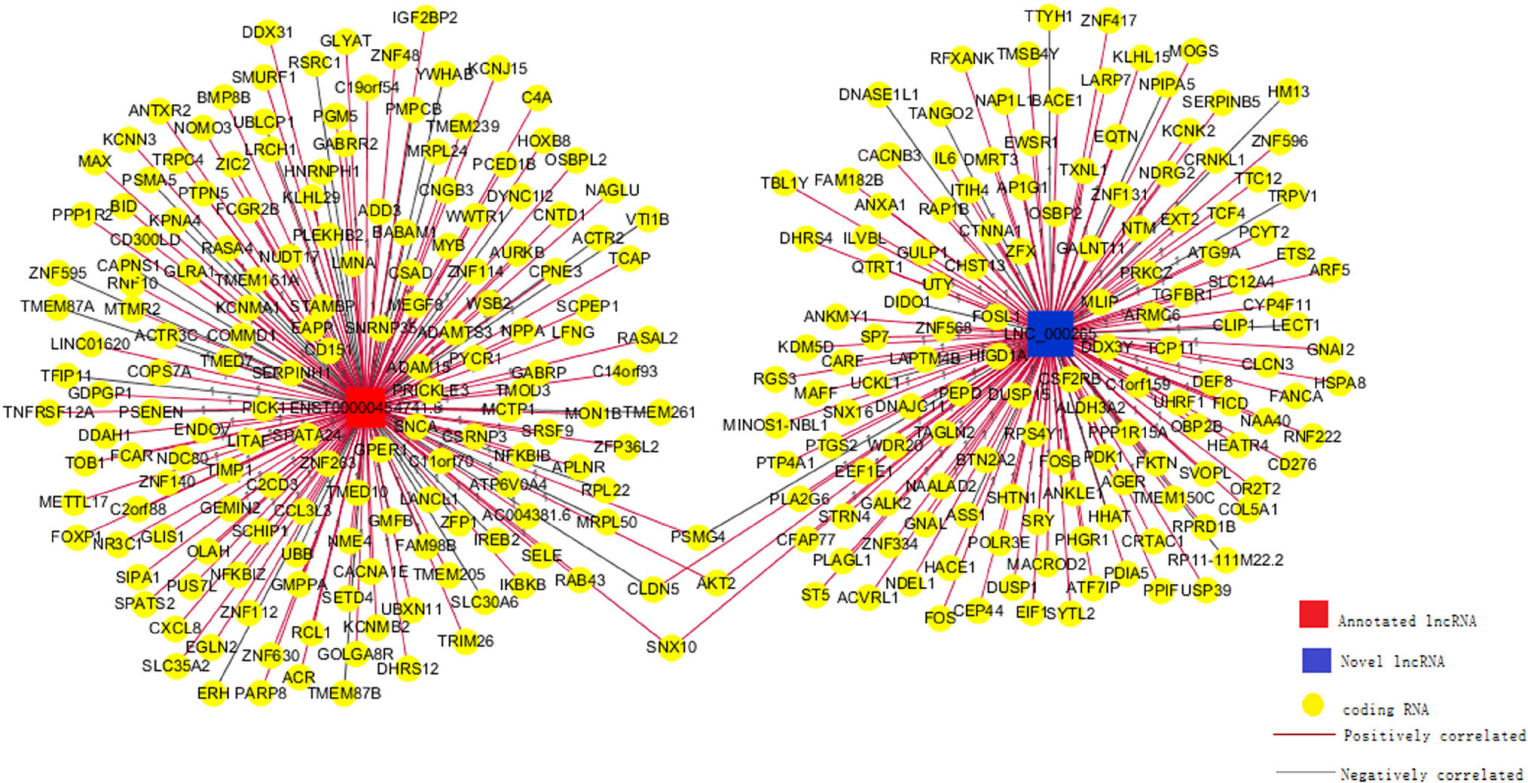
Co-expression network for one annotated and one novel lncRNA Two selected lncRNAs were co-expressed with 318 coding gene transcripts. The expression of LNC_000265 correlated with 153 coding gene transcripts (Pearson Correlation Coefficient >0.98).

Numerous lncRNAs regulate their target protein-coding genes in a trans fashion. LNC_000265 (LNC_000265, ENST00000454741.5) was selected for further analysis. The *trans*-regulating target mRNAs of LNC_000265 that exhibited a PCC score higher than 0.98 were used to construct the network using the Cytoscape 3.2 software (Figure 9). The connections between lncRNAs and mRNAs were established through nodes and edges on the diagram. The complex network indicates that the same lncRNA can simultaneously control multiple protein-coding genes through antisense regulation, and the same protein-coding gene can further be regulated by multiple lncRNAs concomitantly.

## DISCUSSION

The present study provided a comprehensive bioinformatics analysis regarding the expression of lncRNAs and mRNA transcripts in the skin tissue of subject with SS compared with normal skin. A total of 33 and 950 lncRNAs and mRNAs were up- regulated, whereas 38 and 1565 lncRNAs and mRNAs were down-regulated. Hierarchical clustering analysis revealed that the heat signature of the pathological tissue samples could be distinguished from the normal skin samples, whereas the majority of the genes that were present in enriched pathways were those that participated in focal adhesion, PI3K-Akt signaling, and cancer-related pathways. The data suggest that this analysis can yield hits in the discovery of key target genes involved in the development of SS.

The investigation of the key genes that are involved in SS has not been extensively reported to date possibly due to the lack of a definitive medical diagnostic consensus for this broad category of diseases [33]. Nevertheless, the identification of genes involved in skin diseases that are similar in pathology and can be medically defined has been previously reported. Diseases such as psoriasis, atopic dermatitis, and atopic eczema have been investigated in terms of bioinformatics and qPCR analyses [30, 34–39]. Although evidence regarding the aforementioned diseases from bioinformatics studies is limited, experimental studies have shown the contribution of certain genes in the development of skin pathophysiological diseases, namely genes involved in lipid homeostasis of the skin barrier (CERS4, [35]), genes involved in cell adhesion, migration, endocytosis and skin barrier (SDC-4, [37]), and genes that participate in the inflammatory response (IL-17, IL-20, IL-24, IL-31, and IL-33, [41, 43]. For example SDC-4 (syndecan-4) mRNA levels were increased in atopic dermatitis compared with normal skin samples [37] and the inflammatory mediators IL-17, IL-20, IL-24, IL-31, and IL-33 were also elevated in atopic dermatitis [34, 36]. The aforementioned experimental findings were confirmed by a bioinformatics study that identified 438 and 779 DEGs that were enriched in pathways involved in epidermis development and immune response, respectively [30], whereas a study that used human DNA microarrays revealed similar findings with regard to the participation of DEGs in immune response, lipid homeostasis, and epidermal differentiation in atopic eczema [40]. The present study identified 71 lncRNA transcripts (33 up-regulated and 38 down-regulated) and 2615 mRNA transcripts (950 up-regulated and 1565 down-regulated) that were differentially expressed in skin tissue of subject with SS compared with normal skin, which is in agreement with the studies by Ding *et al*. and Zhang *et al*. that demonstrated similar trends in the number of DEGs [30, 39]. In addition, GO analysis demonstrated that the KRT27 and CLDN5 genes, which are associated with keratinocyte differentiation and epidermal development, were highly expressed in the SS GO term “system development”, whereas IL-27RA and CCL18, which are key cytokine and chemokine genes involved in inflammation, were specifically expressed in the SS GO term “protein binding and binding”. These findings are in concordance with the studies of Ding *et al*., Zhang *et al*., and Nishiyama *et al*., in which the main pathways enriched were those corresponding to the inflammatory response and epidermis development [30, 39, 40].

With regard to the KEGG pathway analysis, the data indicated that the transcripts with the lowest q value were involved in transcriptional misregulation in cancer, cancer-related pathways, focal adhesion, and ECM receptor interactions. Importantly, the PI3K-Akt signaling pathway was also involved. The study by Zhang *et al*. identified a similar target gene, namely PI3KR1 (phosphoinositide-3-kinase regulatory subunit 1) in the chemokine signaling pathway that was the most enriched with regard to DEGs in atopic dermatitis [39]. Moreover, LAMA5, ITGB4, and other protein-coding genes associated with epidermal homeostasis were enriched in both pathways [41, 42]. Certain studies have suggested that the epidermal barrier function of SS is disrupted [43, 44]. Therefore, ECM-receptor interaction signaling and the PI3K-Akt signaling pathway may play an important role in the pathogenesis of SS. Furthermore, it is important to note that the pathways of cancer and inflammation are linked. For example, tobacco smoke acts as a tumor promoter by virtue of its high carcinogenic compound content can further trigger chronic inflammation [45]. Moreover, the majority of solid malignancies that appear in older individuals and cell senescence [46] are postulated to be tumor promoters that act through inflammatory mechanisms. In addition to its pro-tumorigenic effects, inflammation influences the host immune response to tumors and can be targeted to augment the response to chemotherapy [47]. Thus, it may be speculated that pathways involved in cancer may cross-talk with pathways involved in the inflammatory response, which provides an explanation for the results obtained by KEGG analysis in the present study.

Although the aforementioned studies have notably focused on the differential expression of mRNA transcripts, the contribution of lncRNAs in skin-associated diseases remains less studied. Ahn *et al*. demonstrated [31] that more than 50% of coexpressed genes, identified in three network modules that correlated with psoriasis and six network modules that correlated with biological treatment, were lncRNAs [31]. The current study identified the lncRNA LNC_000265 by RNA-seq and qRT-PCR analysis. This lncRNA was used to construct networks with genes that could be potentially correlated with regard to their transcripts. LNC_000265 and its predicted *trans*-regulated target CLDN5 were highly correlated. The protein Claudin5 encoded by CLDN5 plays a vital role in the epidermal barrier structure of tight junctions (TJs). TJs are intercellular contacts that seal the space between the individual cells of an epithelial sheet so as to collectively separate tissue compartments. In comparison with other junctions, TJs are composed of approximately 40 different proteins including claudins (Cldn) and zonula occludens (ZO), which are known to be important for barrier function in epithelial cells [48]. The data indicated that CLDN5 was expressed at low levels in skin tissue of subject with SS compared with normal skin (*P* < 0.05, log2FoldChange = −1.93758). Furthermore, LNC_000265 was closely related to its predicted *trans*-regulated target CLDN5 (PCC= 0.98). The results of the qRT-PCR assay demonstrated that LNC_000265 and CLDN5 were also expressed at low levels in skin tissues of subjects with SS. This gene cluster further included CLDN1, CLDN2, CLDN6, and CLDN12, and exhibited a strong correlation with TJs [49]. The findings suggest that LNC_000265 may play a role in the epidermal barrier structure of TJs by regulating the expression level of CLDN5.

Despite the aforementioned findings that expand the existing knowledge on the molecular gene network interactions that are observed in SS, the present study has several limitations. Although the data presented were consistent with previously published reports, the overall sample size was considerably small and only females were included, which could induce bias in the conclusions of the study. Additional bias is caused by the application of a gene set enrichment analysis that is based on the rational that gene networks are constructed based on network or pathway information. In contrast to gene set enrichment analysis, weighted gene coexpression network analysis (WGCNA) can be used in future studies to prioritize the main genes in a given network by the estimation of the connectivity of each gene as it has been noted by previous studies [31]. A WGCNA-based screen can achieve a higher validation rate compared with a differential expression analysis in biomarkers of glioblastoma [49]. Furthermore, the present study used sensitive skin tissue, in the absence of the isolation of specific cell types from the tissues. Future studies are needed to address the contribution of each gene network to a particular cell type, whereas the verification of the target genes can be further validated by the use of appropriate KO cell and animal models. Finally, no direct experimental evidence was provided to confirm the results of lncRNA function prediction. Our future research will involve the construction of appropriate cell models in order to address these limitations.

In conclusion, the present study provided a comprehensive analysis of the DEGs with regard to lncRNA and mRNA transcripts in skin tissue of subject with SS compared with normal skin by RNA-seq and bioinformatics analysis. This study indicated that lncRNAs contribute a critical role in the pathogenesis of SS, which may aid the identification of the pathogenesis of SS and the development of potential targets for personalized therapeutic strategies.

## METHODS

### Subjects and samples

A total of 26 patients who were scheduled to undergo facial nevus resection surgery at the dermatology department of the First Affiliated Hospital of Kunming Medical University were enrolled. The skin sensitivity was evaluated according to a questionnaire (Table 4) [50] and a lactic acid stinging test [51]. The subjects that responded positive to a minimum of 5 out of 7 questions and were associated with a stinging test score greater than or equal to 3 were classified as SS. The exclusion criteria were defined as the incidence of SS that resulted from acne, pityriasis rosea, contact dermatitis, eczema, and/or other skin diseases (Table 5). The skin tissue was obtained by trimming around the nevus. The tissue was mixed with RNA-later solution (QIAGEN, Germany) and stored at −80°C. The study protocol was approved by the Ethics Committee Board of the First Affiliated Hospital of Kunming Medical University. Informed written consent was obtained from each participant.

**Table 4:** Questionnaire for diagnosis of SS

1	Would you say that your face/neck does not tolerate cold/hot weather or a cold/hot environment?
2	Would you say that your skin face/neck does not tolerate rapid temperature changes?
3	Have you already avoided the use of some cosmetic products that could, according to you, make your skin reactive?
4	Have you already had an adverse reaction on your face/neck to a cosmetic or hygiene product?
5	Would you say that your face/neck is reactive?
6	Have you already felt some itching, burning or tingling on your face/neck skin because of the wind or some cosmetics or hygiene products?
7	Is your face skin reactive to pollution, stress/emotions or menstrual cycle changes?

**Table 5:** Summary of subjects characteristics

Characteristics	Sensitive skin (SS)	Normal skin (N)
**Total number**	**13**	**13**
Gender	Female	Female
Age (y), mean ± SD	38.5 ± 7.7	38.0 ± 7.6
Results of questionnaire, mean ± SD*	6.3 ± 0.8	1.0 ± 0.7
Scores of lactic acid stinging test, mean ± SD*	4.9 ± 0.9	1.6 ± 0.2
**RNA sequencing**	3	3
Age (y), mean ± SD	40.3 ± 6.7	39.3 ± 8.1
**qRT-PCR**	10	10
Age (y), mean ± SD	38 ± 8.2	37.6 ± 7.9

^*^P < 0.05 were considered significant by *t*-test

### Library preparation and sequencing process

Total RNA was extracted from the pathological samples (*n* = 3) and the normal skin samples (*n* = 3). The extraction was carried out using TRIzol reagent (Invitrogen, USA) according to the manufacturer’s instructions. The RNA quality was assessed using a Nano Photometer spectrophotometer (IMPLEN, CA, USA). The RNA concentration was measured using the Qubit RNA Assay Kit and the Qubit 2.0 Flurometer (Life Technologies, CA, USA). The RNA integrity was verified on an Agilent Bioanalyzer 2100 (Agilent Technologies, Palo Alto, CA). The RNA library was constructed using a total amount of 3 μg of RNA per sample with an RNA integrity number (RIN) over 7.0. The Epicentre Ribo-zero rRNA Removal Kit (Epicentre, USA) was used to remove Ribosomal RNA (rRNA) according to the manufacturer’s protocol. Finally, the dUTP method was used to obtain strand-specific sequencing libraries by the NEB Next Ultra Directional RNA Library Prep Kit for Illumina (NEB, USA), according to the instructions provided by the manufacturer. The RNA-seq assay was carried out on an Illumina Hiseq 2000 platform and 100 bp paired-end reads were obtained. The preparation of the total transcriptome libraries was conducted by Novogene Bioinformatics Technology Cooperation (Beijing, China).

### Reads mapping and transcriptome assembly

The filtered reads were compared with the human genome (hg19) using the TopHat2 software (v2.0.9) [52]. Based on the mapping results derived by TopHat2, the transcriptome was assembled and quantitatively analyzed by the Cufflinks software (v2.1.1) [53].

### Differential expression analysis

Sequenced reads (raw data or raw reads) in the FASTQ format were first processed via in-house Perl scripts. Clean reads were prepared by deleting reads containing ploy-N, adapter, and low quality reads from raw data. GC content, Q20, and Q30 of the cleaned data were calculated. The false discovery rate (FDR) was calculated as described previously [54] and a FDR of 1% was used. Clean reads were mapped to the reference genome built with Bowtie (v2.0.6) [55]. The mapped reads were assembled by Cufflinks (v2.1.1) in a reference-based approach [53]. The DEGs were identified at a confidence interval of 95% for each experiment. The alternative splicing (AS) events were analyzed using rMATS (http://rnaseq-mats.sourceforge.net/index.html). In the case of a comparison group with a differential variable splice analysis, we first counted the types and quantities of variable splice events, and then calculated the amount of variable splice events for each category, and finally for each variable splice events for differential analysis.

### Gene Ontology (GO) and Kyoto Encyclopedia of Genes and Genomes (KEGG) enrichment analysis

Differentially expressed transcripts were analyzed by GO enrichment and KEGG pathway in order to identify possible enrichment of genes with specific biological pathways (http://www.genome.jp/kegg/pathway.html). GO (http://www.geneontology.org/) is a database that provides vocabularies and classifications associated with the molecular and cellular structures and functions regarding the biological annotations of genes [56]. GO terms consist of three categories: biological processes (BP), cellular component (CC), and molecular function (MF). The KEGG (http://www.genome.jp/kegg/ or http://www.kegg.jp/) database includes information regarding the contribution of DEGs in known metabolic pathways and regulatory pathways, and facilitates the mapping of genes to KEGG pathways for systemic analysis of gene function. GO and KEGG pathway enrichment analysis were conducted for the upregulated and downregulated DEGs, respectively. KEGG analysis was used to see in which pathways the DEGs participated. If multiple transcripts are enriched in a specific pathway, the *q* value for the enrichment of the pathway was calculated, rather than the q value of each transcripts.

GO enrichment was performed with the GO Seq R package, with GO terms with *P* < 0.05 being considered as significantly enriched. The KOBAS (KO Based Annotation system) software (Beijing, China) was used to assess statistical enrichment in KEGG pathways. GO enrichment indicates the relationship between genes and GO terms. For each gene *g* and each GO term GO*j*, a score is generated, which is typically referred to as the gene ontology enrichment score. The enrichment score refers to the ratio of the number of genes located in the pathway entry in the differentially expressed genes to the total number of genes in the annotated gene located in the pathway entry (or “enrichment factor” = “the number of candidate genes”/”the number of background genes”). The greater the enrichment score, the greater the degree of enrichment. The *q* value is the value of the *P* value after multiple hypothesis test correction.

### Prediction of the function of lncRNAs

The function of most LncRNAs in current databases is unclear. The biological functions of lncRNAs were analyzed through the position of target protein coding genes (cis) and expression correlation (trans). We set the co-location threshold to lncRNA upstream and downstream 100 kb, and analyzed the co-expression pairs by calculating Pearson correlation coefficients (PCC).

### Quantitative real-time PCR (qRT-PCR)

Total RNA isolated from 10 SS patients and 10 normal facial tissues using Trizol reagent (Invitrogen, USA) was reverse transcribed to cDNA using All-in-One First- Strand cDNA Synthesis Kit (Gene Copoeia, USA), according to the manufacturer’s protocol. Two significantly upregulated (ENST00000441722.5 and ENST00000624863.1) and two downregulated lncRNAs (LNC-000265 and ENST00000414790.5) were selected to test the reliability of the analysis. In addition, one mRNA transcript (ENST00000413119) was selected to validate the result. The 2^−ΔΔCt^ method was used with β-actin as an internal control in order to relatively quantify the detected transcripts. The primer sequences are listed in Table 6.

**Table 6:** Primer pairs used for qRT-PCR experiments

Gene ID	Primer sequence
ENST00000413119	Forward 5′- CCGAGTCGTACACTTTGCAC -3′
	Reverse 5′- CCTTCCTGGACCACAACATC -3′
ENST00000441722.5	Forward 5′- CAGTCAGGAGAAAGAAGTGGAGG -3′
	Reverse 5′- ACAAATAAGAGGGGACAGAGGTG -3′
ENST00000624863.1	Forward 5′- GGATGGGAGACAAGCATAGAAAAT -3′
	Reverse 5′- TGTGAGGAGACCTGGTATAGAAAC -3′
ENST00000414790.5	Forward 5′- ACGAGTGTGCGTGAGTGTGAG -3′
	Reverse 5′- ATTGATGATGAGTCCAGGGCT -3′
LNC-000265	Forward 5′- CCTTCCCTGATGTCTGATTTTTG -3′
	Reverse 5′- GCCTCTTCTCCCATTTGTTTTTC -3′
β-Actin	Forward 5′- CCAGGGCGTTATGGTAGGCA -3′
	Reverse 5′- TTCCATATCGTCCCAGTTGGT -3′

## SUPPLEMENTARY TABLES






