# Supplementary material for: Genome-wide identification of long non-coding RNA and mRNA profiling using RNA sequencing in subjects with sensitive skin
Source: Oncotarget. 2017 Dec 12;8(70):114894–910. doi: 10.18632/oncotarget.23147 (PMC5777740; doi:10.18632/oncotarget.23147)
Supplement: Supplementary file 1 [file oncotarget-08-114894-s001.pdf]

Genome-wide identification of long non-coding RNA and mRNA profiling using RNA sequencing in subjects with sensitive skin

SUPPLEMENTARY MATERIALS

Supplementary Table 1: The information of annotated lncRNAs. See Supplementary_Table_1

Supplementary Table 2: The GO enrichment result of differentially expressed mRNAs. See Supplementary_Table_2
